# Exercise and Caloric Restriction Alter the Immune System of Mice Submitted to a High-Fat Diet

**DOI:** 10.1155/2013/395672

**Published:** 2013-03-13

**Authors:** Frederick Wasinski, Reury F. P. Bacurau, Milton R. Moraes, Anderson S. Haro, Pedro M. M. Moraes-Vieira, Gabriel R. Estrela, Edgar J. Paredes-Gamero, Carlos C. Barros, Sandro S. Almeida, Niels O. S. Câmara, Ronaldo C. Araujo

**Affiliations:** ^1^Department of Biophysics, Federal University of São Paulo, 04023-062 São Paulo, SP, Brazil; ^2^School of Arts, Sciences and Humanities, University of Sao Paulo, Avenue Arlindo Bettio 1000, 03828-000 São Paulo, SP, Brazil; ^3^Department of Immunology, Laboratory of Transplantation Immunobiology, Institute of Biomedical Sciences, University of São Paulo, 05508-900 São Paulo, SP, Brazil; ^4^Department of Nutrition, School of Nutrition, Federal University of Pelotas, 96010-610 Pelotas, RS, Brazil

## Abstract

As the size of adipocytes increases during obesity, the establishment of resident immune cells in adipose tissue becomes an important source of proinflammatory mediators. Exercise and caloric restriction are two important, nonpharmacological tools against body mass increase. To date, their effects on the immune cells of adipose tissue in obese organisms, specifically when a high-fat diet is consumed, have been poorly investigated. Thus, after consuming a high-fat diet, mice were submitted to chronic swimming training or a 30% caloric restriction in order to investigate the effects of both interventions on resident immune cells in adipose tissue. These strategies were able to reduce body mass and resulted in changes in the number of resident immune cells in the adipose tissue and levels of cytokines/chemokines in serum. While exercise increased the number of NK cells in adipose tissue and serum levels of IL-6 and RANTES, caloric restriction increased the CD4+/CD8+ cell ratio and MCP-1 levels. Together, these data demonstrated that exercise and caloric restriction modulate resident immune cells in adipose tissues differently in spite of an equivalent body weight reduction. Additionally, the results also reinforce the idea that a combination of both strategies is better than either individually for combating obesity.

## 1. Introduction

Chronic, low-grade inflammation is associated with insulin resistance, type 2 diabetes, and several types of cancer [1, 2]. These detrimental conditions are also associated with obesity [3]. As visceral and subcutaneous adipocytes increase in size, monocytes and CD4+ and CD8+ T cells migrate to adipose tissue (AT) [4] initiating the release of proinflammatory mediators (e.g., IL-1*β*, IL-6, RANTES, MCP-1, and IL-18) inducing local insulin resistance [5]. Thus, the expanded ATs and their populations of resident immune cells constitute the main microenvironment in which proinflammatory cytokines are produced and released in the organism [6]. 

Physical activity and caloric restriction (CR) are both nonpharmacological strategies recommended to reduce obesity [7]. Although the beneficial effects of exercise are well described in skeletal muscle and the liver, the same is not true for AT [7–9]. Additionally, there is little information concerning the effects of both weight reduction strategies on immune cell populations that reside in AT. In relation to CR, though its importance in reducing body weight is unquestionable, consumption of a healthy diet requires such marked lifestyle changes that many individuals are unable to comply with one of them.

Thus, it is important to investigate whether physical exercise and CR are able to promote a healthy lifestyle while maintaining a high-fat (HF) diet. The aim of this study was investigate the effect of physical exercise or CR on AT immune cells in diet-induced obese mice.

## 2. Materials and Methods

### 2.1. Animals

Male C57BL/6 (*n* = 20, 5 per group) mice (aged 8–12 weeks; 23–26 g) were obtained from the Animal Care Facility at the Federal University of São Paulo (UNIFESP). All animals were housed in standard, individual cages and had access to water and food. To examine the changes in stromal vascular cell populations in adipose tissue under conditions of diet-induced obesity, we divided the C57BL/6 mice into four groups and fed them either a standard chow diet (6% fat, Nuvilab mod. CR-1) or a high-fat diet (D12451, 45% Kcal fat, Research Diets). At 16 weeks, the mice were further subdivided into the following groups: (1) a control group fed a normal, low-fat (LF) chow; (2) a control group fed a high-fat (HF) diet; (3) a dietary restriction group fed a 30% high-fat diet (HFREST); and (4) an exercise group fed a high-fat diet that participated in 60 minutes of swimming (HFEX). Food consumption was controlled every day. Based on the quantity of high-fat diet chow consumed and using of the macronutrient composition as reference, we calculated the energy intake. The 30% caloric restriction was designed taking HF consumption as a reference. 

All procedures were previously reviewed and approved by the internal ethical committee of the institution.

### 2.2. Exercise Protocol

The HFEX animals were subject to swimming sessions in a swimming system adapted for mice with water heated to 30°C. The 300 liter tank had 10 lanes and was fitted with air pumps that maintained the mice in constant motion. Swimming sessions began with 15 minutes in the first week and gradually increased in length until the mice were able to swim for 60 minutes a day. At this point, the HFEX mice were subjected to swimming sessions 5 times per week for 6 weeks. Both the exercise and the dietary restriction groups were subject to their respective intervention for 6 weeks.

The mice were anesthetized with ketamine/xylazine for blood collection via retroorbital venous plexus and then killed by cervical dislocation. The blood was centrifuged at 1000 g for 10 minutes. The serum was removed and stored at −80°C for future analysis. We collected a 1 g sample of adipose inguinal from each group and subjected the sample to enzymatic degradation. All animals were weighed weekly until the end of the experiments.

### 2.3. Isolation of the Stromal Vascular Fraction (Sfv) and Flow Cytometry

After sacrificing the mouse, inguinal adipose tissue (IAT) was extracted, weighed, and subjected to enzymatic degradation as previously described [10]. After the isolation of the IAT SFV cells, 200 *µ*L of FCS washing buffer (1x PBS, 2% SFC) was added, and the solution was centrifuged for 5 minutes. After the supernatant was discarded, the pellet was resuspended in FCS and centrifuged for 5 minutes at 600 g. The cells were stained with anti-CD8 (Caltag-FITC-Medsystems, Buckingham, UK), anti-CD4 (blue-Pacific-BioLegend), anti-F4/80 (PerCP-Bioscience), and anti-NK.1 (PE-Bioscience) antibodies. The stromal cells were acquired via FACS in a Canto II flow cytometer (BD, Becton Dickinson, NJ, USA). The data analyses were completed using the program FlowJo 8.7.4. (Tree Star Inc., Ashland, OR, USA). 

### 2.4. Analysis of Cytokines in Serum

Serum samples were stored at −80°C. The panel used for the Milliplex Mouse cytokine/chemokine immunoassay included the following cytokines: MCP-1, RANTES, TNF-alpha (tumor necrosis factor), IL-6, and IL1-*β*. Testing was conducted in accordance with the procedures previously described by the manufacturer (Milliplex Mouse cytokine/chemokine panel).

### 2.5. Glucose Tolerance Test

The glucose tolerance test (GTT) was carried out in animals fasted for 12 hours. To avoid stress, there was an interval of 7 days between tests. Glycemia was measured using a glucometer (Accu-Chek Advantage) measuring blood drops obtained from the tail vein. For GTT 1 g glucose per kg of body weight (BW) was injected intraperitoneally. Glucose levels were determined at baseline, 0, 15, 30, 60, and 120 min after the injection of glucose.

### 2.6. Statistical Analysis

The data were presented as the mean ± standard error in the descriptive text and graphics. All experiments were compared using One Way ANOVA followed by post-hoc Tukey test. Significant differences were determined when the *P* value was less than 0.05 (*P* < 0.05). The graphics were developed in Prism 5.0.

## 3. Results

**Figure 1:**
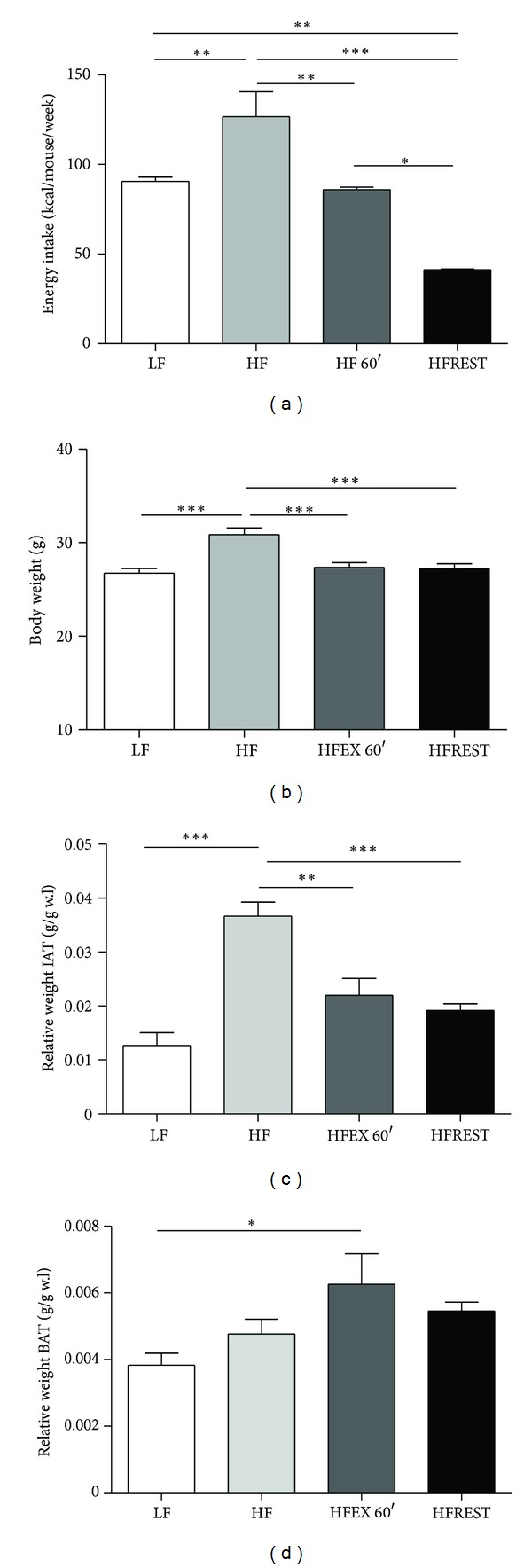
(a) Weekly caloric intake of animals subjected to the control diet (LF), high-fat diet (HF), high-fat diet with exercise (HFEX 60′), and high-fat diet with 30% food restriction (HFREST). (b) Mouse body weights (g). (c) Relative weight of IAT (inguinal adipose tissue). (d) Relative weight of BAT (brown adipose tissue). Mean ± SEM, *n* = 5 mice per group. ***P < 0.001, **P < 0.01, *P < 0.05.

Animals subjected to the HF diet consumed more calories compared with mice from the LF diet group (Figure 1(a)). Higher caloric consumption was accompanied by an increase in mouse total body mass (Figure 1(b)). Swimming combined with the HF diet was able to reduce mouse body weight similar to that observed in animals administered caloric restriction (Figure 1(b)). There was no difference in inguinal adipose tissue (IAT) and brown adipose tissue (BAT) between trained animals and those submitted to caloric restriction (Figures 1(c) and 1(d)); however, trained animals presented more BAT than animals from LF group (Figure 1(d)). 

**Figure 2:**
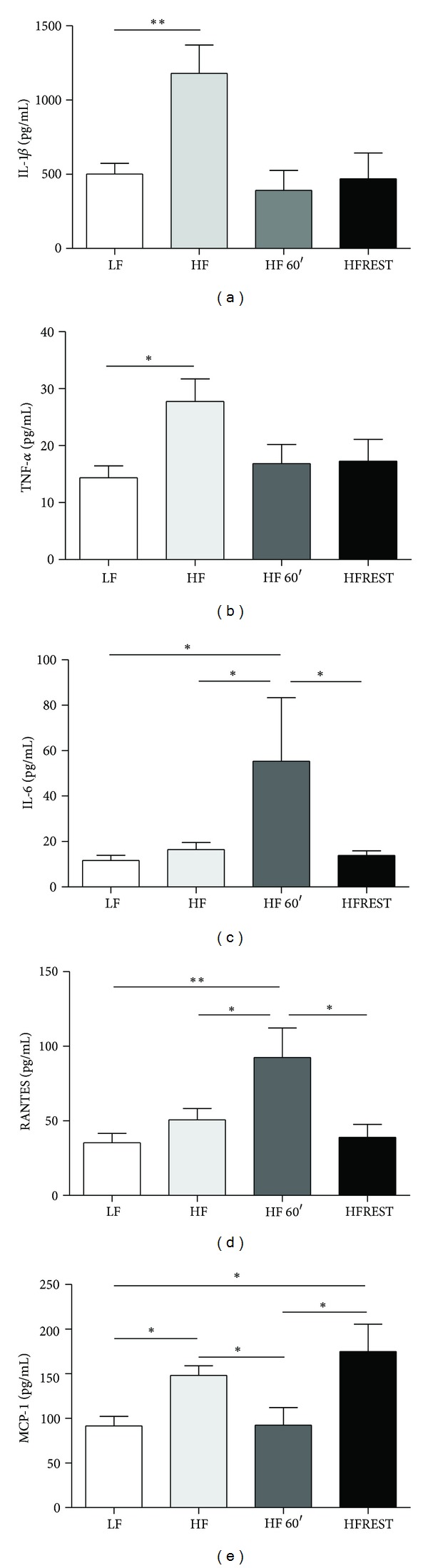
Cytokine concentrations (pg/mL) in mouse serum subjected to control diet (LF, *n* = 12), high-fat diet (HF, *n* = 12), high-fat diet with exercise 60′ (HFEX 60′, *n* = 8), and high-fat diet with 30% food restriction (HFREST, *n* = 8). (a) IL-1*β*, (b) TNF-*α*, (c) IL-6, (d) RANTES, and (e) MCP-1. Mean ± SEM. ***P < 0.001, **P < 0.01, *P < 0.05.

It is known that obesity is associated with systemic, low-grade inflammation. Therefore, we investigated the effects of a HF diet, exercise, and diet restriction in our study groups by evaluating the serum levels of several proinflammatory cytokines. We observed increased levels of IL-1*β* in the HF diet group and a reduction of this cytokine in both intervention groups (exercise and caloric restriction) (Figure 2(a)). TNF-*α* serum levels were not affected by the changes in diet and exercise investigated in this study (Figure 2(b)). 

In addition to exerting both pro- and anti-inflammatory functions, IL-6 also plays important roles in both obesity and physical exercise. While the HF diet did not change IL-6 levels, exercise training increased its levels (Figure 2(c)). Reduction of body weight was unable to induce changes in IL-6 levels as no differences were observed in animals from the caloric restriction group (Figure 2(c)).

Animals subjected to swimming had significantly increased RANTES serum levels (regulated upon activation, normal T cells expressed and secreted). The increases in body mass and IAT observed in animals submitted to the HF diet did not lead to changes in RANTES levels, albeit AT lymphocyte infiltration during obesity is expected. Nevertheless, physical exercise increased RANTES serum levels, while caloric restriction did not change its levels (Figure 2(d)). Moreover, MCP-1 (monocyte chemoattractant protein-1) was reduced by exercise and was not affected by caloric restriction (Figure 2(e)). 

**Figure 3:**
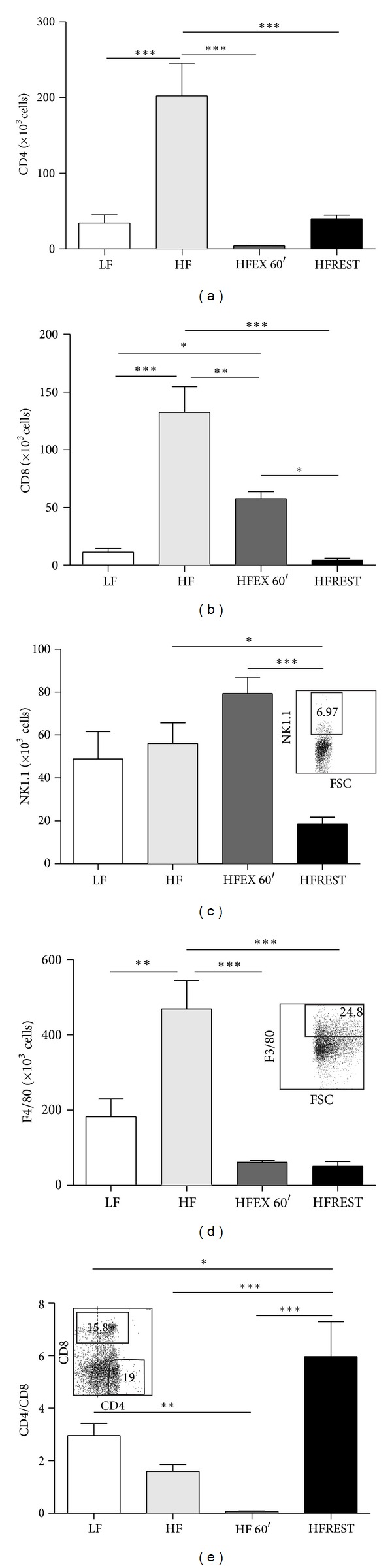
Quantification of cells expressing CD4, CD8, NK1.1, and F4/80 (macrophage) in inguinal adipose tissue of mice subjected to control diet (LF), high-fat diet (HF), high-fat diet with exercise (HFEX 60′), and high-fat diet with 30% food restriction (HFREST). (a) CD4 cells, (b) CD8 cells, (c) NK1.1 cells, (d) F4/80 cells, and (e) ratio of CD4/CD8. Mean ± SEM, *n* = 5 mice per group. ***P < 0.001, **P < 0.01, *P < 0.05.

Because immune cells that reside in obese AT actively secrete proinflammatory cytokines and chemokines, we evaluated the effect of the HF diet and both interventions on AT leukocyte populations. We observed a reduction in CD4+ and CD8+ T lymphocytes in AT in animals submitted to both interventions in comparison with animals consuming the HF diet (Figures 3(a) and 3(b)). Because CD8+ cells were more reduced than CD4+ cells, an increased CD4+/CD8+ ratio was observed (Figure 3(e)). The natural killer cell marker (NK1.1) was affected only by caloric restriction (Figure 3(c)). Also, we observed that both exercise and caloric restriction were able to reverse the increased macrophage infiltration observed in the HF diet group (Figure 3(d)). 

**Figure 4:**
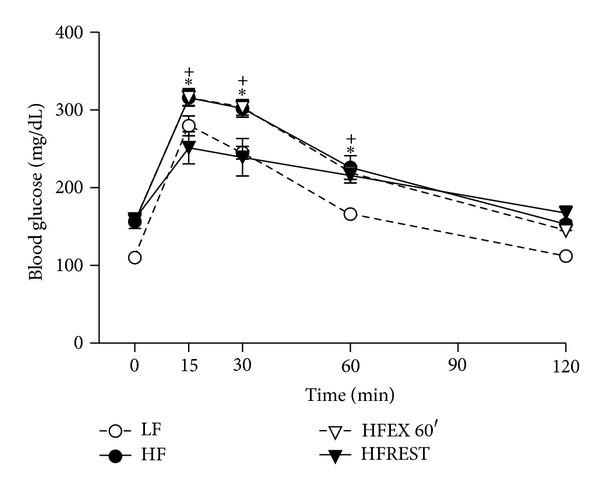
Glucose tolerance test in mouse subjected to control diet (LF), high-fat diet (HF), high-fat diet with exercise 60′ (HFEX 60′), and high-fat diet with 30% food restriction (HFREST). The glucose levels of LF were different from the other groups at time 0, 30, and 60 minutes (*P* < .05). Mean ± SEM, *n* = 5 mice per group (^+^ *P* < 0.05, HFEX 60′ versus HFREST; *P < 0.05, HF versus HFREST).

Concerning glucose tolerance test, it was observed that only caloric restriction was able to improve this parameter in comparison to HF group (Figure 4).

## 4. Discussion 

Immune cells reside in lean and obese adipose tissue but exhibit different characteristics in each condition. As adipocytes increase in size, these immune cells change in terms of number and functionality. Moreover, such changes in immune cells contribute actively to the establishment of local and systemic low-grade inflammation [11]. Regular exercise is an important nonpharmacological strategy for treating obesity as it protects against the increases in body mass and counterbalances several deleterious consequences due to its anti-inflammatory effects [12]. Normally, exercise intervention with dietary modifications is suggested to combat obesity; the combination of both interventions works better than exercise alone [13–15]. However, little is known about the benefits of exercise when dietary modifications are not prescribed. 

Because our results demonstrated that chronic exercise is able to counterbalance several immune changes induced by HF (or promotes different changes), it appears that this intervention is beneficial even when a high-fat diet is maintained. It was previously demonstrated that AT expansion reduces the number of resident NK cells. NK cells produce significant amounts of gamma interferon (IFN-*γ*), promoting an inflammatory state in AT. Studies have also shown that reducing the presence of inflammatory cells in AT improves glucose tolerance in IFN-*γ*-deficient mice [16]. IFN-*γ* is also able to inhibit the Hedgehog signaling pathway involved in adipocyte differentiation [17]. The ability of exercise to promote an increase in the number of NK cells in AT may also reflect another type of cell infiltration, such as NKT, that appears to display a protective role. The absence of changes in the number of NK cells corroborates with two previous studies which reported that loss of NK cells has little or no effect on metabolic parameters after a 45% HF diet for 26 weeks or 60% of the same diet for 12 weeks [18, 19]. 

In concordance with previous results [20, 21] we observed an increased frequency of macrophages in obese AT. Macrophages are responsive to TLR stimuli producing marked amounts of proinflammatory cytokines (e.g., IL-12, TNF, IL-1*β*, and IL-6) increasing AT inflammatory response [22]. Additionally, these immune mediators are involved in insulin resistance and type 2 diabetes in obese organisms [23]. Thus, our data regarding macrophages and adipose tissue from the HF diet animals were in accordance with previous studies that showed that an increased infiltration of macrophages in AT was observed. Although we did not evaluate the macrophage profile (e.g., M1 and M2) in AT, we verified that the increase in macrophage number and MCP-1, an important molecule in the recruitment of these cells [24], promoted by HF, was reversed by exercise.

In this study, increased macrophage infiltration due to HF was followed by an increased number of CD8+ T cells in the AT. Conversely, macrophage reduction promoted by exercise and caloric restriction was followed by a decreased number of CD8+ T cells. Adaptive T cells are also related to macrophage infiltration in AT [25]. CD8+ T cells increase 3 to 4 times in the AT of humans and animals subjected to high-fat diets and produce large amounts of cytokines and chemokines. Nishimura and et al. [25] reported that CD8+ T cell neutralization reduced macrophage infiltration and insulin resistance in mice fed with a high-fat diet. The adoptive transfer of CD8+ T cells to mice deficient in this cell population aggravates inflammation in the AT. Together, these data suggest that CD8+ T cells are activated in the AT of obese mice and that these lymphocytes induce macrophage activation and migration to AT. 

CD4+ T cells also play a pivotal role in the progression of obesity and are associated with inflammation via cytokine secretion. In Rag-1 KO mice, reconstitution of CD4+ T cells reduced the increment in body weight, adipocytes size, glucose tolerance, and insulin signaling [26, 27]. In this sense, it is tempting to speculate that the increase of CD4+ T cells due to a HF diet could compensate for the increased inflammation in AT. The reduction of these cells induced by exercise and caloric restriction suggests that inflammation improvement in AT induces a reduction in CD4+ T cells. 

In the blood as well as in most tissues, the CD4+/CD8+ T-cell ratio is generally greater than 2 to 1. HF consumption induced a reduction in this ratio in AT. It is important to note that only caloric restriction was able to restore the CD4+ and CD8+ numbers to the levels observed in LF group, and this could be related to the different responses observed in the glucose tolerance test.

Since immune cells that reside in adipose tissue are an important source of proinflammatory cytokines and chemokines in obesity, we decided to investigate whether an HF diet and interventions affected cytokines. The HF diet reduced MCP-1 and increased RANTES in serum compared with the control. RANTES is a potent chemoattractant for several cell types [28], including NK cells [29]; its increase in the serum of control animals could be due to the higher number of NK cells in these animals. 

Interleukin-6 is a widely studied cytokine in exercise. It has been demonstrated to be increased by up to 100 times in exhaustive acute exercise promoted by skeletal muscle glycogen depletion [12]. Investigations on the effects of chronic exercise on IL-6 revealed that physical training reduces its levels. Our data, in opposition, demonstrated that chronic swimming increased IL-6. Higher increases in IL-6 levels, in response to exercise, have been associated with the reduction of glycogen stores [12]. In our study, mice were subjected to an HF diet during initial swimming training, and this diet was maintained throughout the study. This difference in study design could explain the discrepancy among our data and those from other studies that investigated the effect of chronic exercise on IL-6 levels. Our results show that the anti-inflammatory effects of exercise are present regardless of whether the exercise promotes body mass reduction [12]. In our study, all the changes promoted by exercise were accompanied by a reduction in body mass, AT, and caloric intake. Interesting, it seems that these reductions were not the only factor in determining the changes in AT cell populations or serum cytokines. Although exercise and caloric restriction have both induced a reduction in body mass and AT mass, the biological repercussions of both interventions were different. For example, fewer NK and CD4+ cells were observed in response to changes in diet as compared to exercise. 

Additionally, the effects of dietary restriction on MCP-1, RANTES, and IL-6 levels were different in comparison to those observed in the trained group. These observations suggest that exercise and caloric restriction, thought to be able to attain the same goal, proceed by different mechanisms [30]. Another example of this statement is reduction of food intake promoted by exercise. In accordance with previous studies [31, 32], such effect could be related to the effect of exercise on leptin sensitivity on central nervous system. Trained animals also presented an enhancement of BAT in comparison to LF group and it is tempting to speculate that a change in thermogenesis could influence body weight reduction in trained animals. Therefore, our results suggest that the effect of chronic exercise was not restricted to caloric expenditure due exercise practice. 

A few limitations must also be discussed. Because the changes we observed in the levels of circulating cytokines were not strictly related to the local number of resident immune cells, it is important to note that the absence of detection experiments of adipose tissue cytokines constitute a limitation of our study. Also, note that exercise intensity is an important factor for adaptations to occur. Thus, other different intensities from that which we evaluated could induce different immune changes from those observed herein, and this must be considered as a limitation of our design.

## 5. Conclusions

Our data demonstrate that both exercise and caloric restriction were able to counterbalance the deleterious effects induced by an HF diet. The interventions induced a reduction in body mass and body fat. However, these reductions could not explain all the results because the effects of dietary restriction and exercise were not the same. The exercise appears to affect innate immunity (i.e., NK1.1), while dietary restriction influenced adaptive immunity (i.e., CD4+/CD8+ ratio). Both interventions affected cytokine and chemokine levels in different manners.
